# The First Digital Strategy-Based Method for Training of Executive Functions: Impact on Cognition and Behavioral and Emotional Regulation, and Academic Success in Children With and Without Psychosocial Risk

**DOI:** 10.3390/bs15050633

**Published:** 2025-05-06

**Authors:** David Cáceres-González, Teresa Rossignoli-Palomeque, María Vaíllo Rodríguez

**Affiliations:** Department of Languages and Education, Nebrija University, 28248 Madrid, Spain; dcaceresg@alumnos.nebrija.es (D.C.-G.); mvaillo@nebrija.es (M.V.R.)

**Keywords:** executive functions, cognitive training, metacognition, strategy-based training, children, children at psychosocial risk

## Abstract

STap2Go is the first purely digital strategy-based method for the training of executive functions, making its evaluation relevant. This study assesses the effectiveness of this intervention in children with (at risk) and without (no-risk) psychosocial risk, which refers to socio-educational vulnerability, and examines whether its impact differs between groups. A total of 124 children (9–12 years old) were randomly assigned to either an experimental or an active control group. Individual assessments and family questionnaires were administered (FDT, WISC-V, RIST, BRIEF-2). Both groups received a 12-week intervention. The experimental group showed significant improvements in executive functions, processing speed, IQ, academic performance, and emotional and behavioral regulation compared to the controls. Notably, IQ, metacognition, and working memory continued improving at follow-up, suggesting lasting effects. While both groups benefited, the effects were more pronounced in at-risk children, particularly in BRIEF-2 (Inhibition, Metacognition, Behavioral Regulation) and academic performance in mathematics and language. Moreover, the psychosocial risk control group showed a trend toward deterioration over time. The far transfer achieved thanks to digital strategy-based training seems to have a greater effect on at-risk children, and can be used to compensate for their difficulties.

## 1. Introduction

### 1.1. Conceptualization of Executive Functions

Executive functions (EFs) are cognitive processes essential for cognitive control, behavioral regulation, and adaptive problem-solving in novel situations (Diamond, 2006; Zelazo & Carlson, 2012; Frick et al., 2017). These functions include skills such as planning, goal maintenance, impulse control, working memory, and attentional regulation (Pennington & Ozonoff, 1996). Moreover, EFs not only regulate behaviour in neutral contexts, but also play a crucial role in emotionally charged situations, enabling the effective management of cognitive and affective responses (Bailey & Jones, 2019). In this regard, EFs can be conceptualized as self-regulatory mechanisms that contribute to adaptation and goal achievement, overlapping with the concept of ‘effortful control’ (Tiego et al., 2020), which implies the ability to deliberately allocate attentional and cognitive resources in an efficient manner (Doebel et al., 2018; Miyake & Friedman, 2012).

On the other hand, metacognition enables individuals to reflect on their own thought processes. This includes awareness of personal strengths and weaknesses, strategic planning, progress monitoring, and outcome evaluation (Pozuelos et al., 2018; Rossignoli-Palomeque et al., 2020). Metacognitive skills enable children not only to execute cognitive tasks but also to develop a deeper understanding of learning processes, allowing them to optimize their learning strategies (Eberhart et al., 2025).

One of the most recognized models of EFs is the three-dimensional model proposed by Miyake et al. (2000), which has been influential in recent decades (Miyake & Friedman, 2012). This model identifies three independent but interrelated core factors of EFs: inhibition, working memory (WM), and cognitive flexibility. Inhibition refers to the ability to refrain from or interrupt actions, thoughts, and emotions that do not contribute to the task at hand (Diamond, 2013; Tamm & Nakonezny, 2015). It also involves the capacity to postpone immediate gratification (Gagne et al., 2021). WM allows for the manipulation of information in short-term memory, while cognitive flexibility enables shifts in attention, tasks, or strategies to achieve goals (Miyake & Friedman, 2012). Despite the acceptance of the three-dimensional model of EFs, some criticisms suggest a broader dynamic model of EFs. Jones et al. (2016) integrate elements of Miyake et al. (2000) into a broader framework of self-regulation. Doebel (2020) and Perone et al. (2021) understood EFs as goal-directed behaviors that encompass multiple cognitive and emotional elements, including personal experience, knowledge, and feelings.

EFs primarily reside in the prefrontal cortex (PFC) (Miller & Cummings, 2017; Semrud-Clikeman & Bledsoe, 2011; Stuss, 2011), a region particularly developed in humans and responsible for complex functions (Goldberg, 2001). The PFC is one of the last brain regions to mature during development (Fuster, 2001). EF development begins at an early age, with one of the first indicators being the achievement of object permanence (Cassandra & Reynolds, 2005; Isquith et al., 2004). Around the age of two, EFs continue to develop through language and symbol acquisition, which help children guide their behavior (Zelazo et al., 2004; Marcovitch & Zelazo, 2009). This development undergoes critical periods from early childhood to adolescence (Cassandra & Reynolds, 2005; Marcovitch & Zelazo, 2009). Basic EFs, such as inhibitory control, peak between the ages of 12 and 14, while others, such as cognitive flexibility, problem-solving, and WM, continue to develop until approximately 15 to 19 years of age (Cassandra & Reynolds, 2005; Davidson et al., 2006), and even into young adulthood (Larsen & Luna, 2018). The development of EFs depends on PFC maturation (Diamond, 2013), which is influenced by genetics (Friedman et al., 2008) as well as psychosocial elements such as inner speech (Best et al., 2011; Manfra et al., 2014) and metacognition (Roebers & Feurer, 2016). In our study, we test a novel digital EF strategy-based intervention, “STap2Go” (STap2Go, 2022) which incorporates metacognitive strategies. We focused on children aged 9 to 12 because this period is critical for the development of executive functions (EFs). During these years, children improve their ability to manage essential EF tasks, such as planning, working memory, and inhibitory control, which are necessary for academic success and daily functioning (Brocki & Bohlin, 2004; De Luca et al., 2003; Davidson et al., 2006). This age range represents a critical window for EF interventions, ensuring alignment with developmental milestones (Karbach & Kray, 2009).

Executive functions (EFs) are essential across various domains, including social, academic, and professional contexts. Numerous studies have established a correlation between EFs and intelligence (Ardila et al., 2000; Friedman et al., 2006; Rossignoli-Palomeque et al., 2019; Wang et al., 2021), as well as academic performance in key subjects such as reading (Johann et al., 2019; Johann & Karbach, 2020) and mathematics (Best et al., 2011; Cheung & Chan, 2022; Peng et al., 2015).

In addition to their cognitive implications, EFs are crucial for emotional and behavioral regulation (Andrés et al., 2016; Diamond, 2013; Hofmann et al., 2012; Morgan & Lilienfeld, 2000; Riccio et al., 2011; Schoemaker et al., 2012; Séguin, 2009; Séguin et al., 1999). Children displaying higher levels of EFs are shown to excel in the theory of mind, a critical skill for socio-emotional regulation (Cole & Mitchell, 2000; Joseph & Tager-Flusberg, 2004; Sabbagh et al., 2006; Jahromi & Stifter, 2008). This link between EFs and emotional processes may be explained by the fact that emotions are embedded in most daily life challenges (Zelazo et al., 2010). In social contexts, EFs are critical for adapting one’s behavior appropriately (Crick & Dodge, 1994).

Given these relationships, researchers and practitioners can reasonably infer that interventions aimed at enhancing EFs may offer substantial benefits. As a result, several cognitive training programs have emerged in recent decades (Strobach & Karbach, 2021). In our study, we integrate these constructs into our analysis to assess how the STap2Go intervention impacts executive functions, emotional regulation, and academic success. By examining these relationships, we aim to better understand the potential of the intervention in enhancing cognitive and socio-emotional outcomes.

### 1.2. Executive Function Alterations: Children at Psychosocial Risk

The contemporary view of psychosocial risk involves a multi-dimensional, dynamic approach to both risk and protective factors (Martins et al., 2024; Jiménez et al., 2019). Families are considered at psychosocial risk when they are unable to meet their children’s needs, thereby hindering their development and well-being (Rodrigo et al., 2011). Children at psychosocial risk may experience situations such as poverty (e.g., malnutrition, healthcare needs), negative or coercive parenting styles (e.g., exposure to violence or stress), traumatic experiences, or an absence of education (Shore, 1997). Prior studies have examined the effects of material and psychosocial deprivation on children’s health, social competence, academic success, and overall life outcomes (Crawford et al., 2017; Yoshikawa et al., 2012; Engle & Black, 2008).

Psychosocial adversity is associated with deficits in EFs. Research has found poorer EFs in children exposed to early deprivation (Shin & Brunton, 2024), those in low-income environments (Waber et al., 2006), and children in institutional settings (Lewis et al., 2024; Camuñas et al., 2022). Generally, such children exhibit slower development of cognitive control (Hackman et al., 2010; Ison et al., 2015; Lipina et al., 2011) and difficulties in behavioral and emotional regulation (Lozano Gutiérrez & Ostrosky, 2011; Moraine, 2014). They are more likely to experience deficits in mental, motor, and socio-emotional behavior. They tend to present difficulties in peer cooperation and a higher prevalence of ADHD (Sroufe, 2000).

In our study, we compare children without psychosocial risk to those identified as being at psychosocial risk. Our aim is not only to test the effects of STap2Go, but also to study the principle of “compensation accounts”, which posits that those with greater needs benefit more from training (Diamond, 2013; Diamond & Ling, 2019; Karbach & Unger, 2014; Smid et al., 2020). In Canarias (Spain), where our study was conducted, the territorial government has established that children at psychosocial risk include those under the following circumstances: those with basic healthcare needs (e.g., food, housing), school-related needs (e.g., digital shortage, lack of education or school materials), socio-educational needs (e.g., lack of extracurricular activities, lack of stimulation), special educational needs, learning disabilities, and late incorporation into the educational system. Interventions targeting EFs are particularly relevant for this population.

### 1.3. Executive Function Interventions: The Novel Scope of EF Training + Metacognition

Takacs and Kassai (2019) described various digital EF interventions that exhibited “near transfer” (effects in the same domains as those trained, e.g., WM, inhibition, or cognitive flexibility) and/or “far transfer” (effects in other domains not directly trained, such as socio-emotional regulation). The objective of EF interventions should be to achieve both near and far transfer. Given the well-documented relationship between executive functions (EFs) and other cognitive and socio-emotional abilities—such as intelligence, academic achievement, and socio-emotional regulation—our study considers these domains as far-transfer variables. However, meta-analyses of EF training programs frequently highlight a key limitation: the lack of consistent evidence for far-transfer effects and sustained long-term benefits (Melby-Lervåg et al., 2016; Rossignoli-Palomeque et al., 2018; Sala & Gobet, 2019; Simons et al., 2016; Takacs & Kassai, 2019). One possible explanation is that many interventions rely on repetitive practice rather than explicitly teaching strategies that promote the flexible application of EF skills across different contexts (Rossignoli-Palomeque et al., 2018). To address this gap, recent research has begun to explore alternative approaches that integrate EF training with metacognitive strategies—an emerging direction known as “strategy-based training”. Our study examines whether strategy-based training can facilitate far transfer, potentially overcoming this limitation.

This new approach aims to foster deeper, more transferable learning by actively engaging participants in the regulation of their own cognitive processes. It is particularly effective when metacognitive strategies are employed to best engage EF training (Strobach & Karbach, 2021). Metacognition plays a substantial role in EF engagement (Marulis et al., 2020; Chevalier & Blaye, 2016) and EF development (Best et al., 2011; Roebers, 2017). Furthermore, metacognitive strategies are especially beneficial for individuals with lower EFs (Rossignoli-Palomeque et al., 2020). Metacognitive regulation refers to the processes that coordinate cognition (Nelson & Narens, 1994; Reder & Schunn, 2014; Shimamura, 2000), implying self-monitoring processes (Nelson & Narens, 1994). This type of metacognition shares similarities with EF development (Roebers & Feurer, 2016) and facilitates self-regulated learning (Efklides, 2009). Metacognition is even a predictor of intelligence and academic performance (Ohtani & Hisasaka, 2018).

Using digital interventions that incorporate metacognitive strategies represents a new significant scope in education. Precursors of this approach include non-digital “strategy-based training”, in which, in addition to repetitive cognitive tasks, a human instructor provides strategies to the user while the user performs tasks (Morrison & Chein, 2011; Jolles & Crone, 2012). This form of learning support is known as “scaffolding” (Wood et al., 1976).

To our knowledge, only five studies have pursued strategy-based training of EFs, or a combination of digital EF training with metacognition, through randomized controlled studies (Kubota et al., 2023; Partanen et al., 2015) including an active control group (Graziano & Hart, 2016; Jones et al., 2020; Pozuelos et al., 2018). Three of these studies utilized WM training (Cogmed) (Graziano & Hart, 2016; Jones et al., 2020; Partanen et al., 2015), finding greater benefits from WM training combined with metacognitive strategies than from simple WM training alone. In Graziano and Hart (2016), preschool children with behavioral problems who underwent strategy-based training experienced greater gains in academic performance, emotional knowledge, and regulation compared to those who received non-strategy-based training. These benefits were maintained at a 6-month follow-up. In the study of Partanen et al. (2015) on children with special needs, the improvement in EFs lasted longer for those who underwent WM training combined with metacognition (6-month follow-up) than for those who underwent basic WM training. Finally, in typically developing children, gains in WM and mathematical reasoning were greater for those who underwent WM training combined with metacognition (maintained at a 3-month follow-up; Jones et al., 2020).

The other two studies involved all three EF components (Kubota et al., 2023; Pozuelos et al., 2018). In Kubota et al. (2023), children from disadvantaged backgrounds who received EF training combined with metacognition demonstrated greater gains than the active control group in proactive control engagement and EFs. Finally, Pozuelos et al. (2018) tested the combined effect of EF training and metacognition in typically developing preschool children. Children in the metacognitive group showed greater gains in intelligence and significant increases in an electrophysiological index associated with conflict processing. These results suggest that metacognitive scaffolding enhances the influence of process-based training on cognitive efficiency and brain plasticity related to executive attention.

The downside of strategy-based interventions is that they require instructors to undergo specific training in scaffolding (Bosanquet & Radford, 2019), which can be time-consuming and costly. Moreover, the success of the scaffolding relies on the scaffold quality and instructor experience. All training methods applied in these five studies was not entirely digital, as they required a human instructor to provide or supervise the metacognitive strategies (Graziano & Hart, 2016; Kubota et al., 2023; Partanen et al., 2015; Pozuelos et al., 2018), or used metacognitive workbooks in addition to a computer-based program (Jones et al., 2020; Kubota et al., 2023). Regarding the training used in our study, Rossignoli-Palomeque et al. (2020) conducted a randomized active-controlled pilot study with typically developing children. The experimental group experienced significantly reduced attentional and EF problems at follow-up compared to the control group. In this pilot version, the strategies were delivered by an instructor.

STap2Go is the first EF digital strategy-based training that does not require a human instructor. The scaffolding is automated by the system, thanks to a pre-designed decision tree. STap2Go’s metacognitive strategies are directed toward planning, monitoring, and evaluating self-performance. This is why we expect that the STap2Go intervention may impact not only EFs and processing speed, but also IQ, metacognition, academic success, and behavioral and emotional regulation, as presented in the next section. Finally, it seems reasonable to expect stronger EF intervention benefits for children at psychosocial risk compared to children without psychosocial risk (Titz & Karbach, 2014).

### 1.4. Objectives

This study primarily aims to assess the effects of a digital strategy-based method for the training of EFs, “STap2Go”, in children without psychosocial risk (no-risk) and children at psychosocial risk (at-risk) (O1). Specifically, we evaluated Stap2Go’s impact on their EFs, intelligence, processing speed, academic performance, and emotional regulation. We expect that STap2Go will produce near transfer (EFs, processing speed) and far transfer (academic scores, IQ, and emotional regulation) after training for children with and without psychosocial risk. Secondly, we aim to evaluate whether the training impact differs between children without psychosocial risk and those at psychosocial risk (O2). We also anticipate greater training gains for children at psychosocial risk.

## 2. Methods

Following the Declaration of Helsinki, written informed parental consent was obtained from each participant. This study was approved by the ethics committee of Nebrija University (UNNE-2021-012).

### 2.1. Participants

One hundred and twenty-four children aged 9–12 years old participated in this study (M = 11.1, SD = 0.82), with 50.8% being boys and 49.2% girls. The experimenter contacted various schools of Canarias Island (Spain), and ultimately, one of them consented to participate in the study. Children were enrolled in a regular school. Initially, participants were divided into two main groups based on their condition: children at psychosocial risk (at-risk) or children without psychosocial risk (no-risk). Secondly, participants in each condition were randomly allocated to two training groups: the experimental group (STap2Go training) or the active control group. All study participants were informed of the purpose of their participation, and parental consent was obtained from all participants. Parents and teachers were blinded to the children’s group assignments. Table 1 shows participants sociodemographic characteristics. 

The inclusion criteria for the children without psychosocial risk (no-risk) were as follows: (1) aged 9–12 years old, (2) no psychological or psychiatric diagnosis, (3) no special educational needs, (4) no course repetition, (5) an intelligence quotient (IQ) between 85 and 115, (6) and no clinical scores on the Behavioral Assessment of Executive Function (BRIEF-2) behavioral and emotional regulation indices at T1 (Gioia et al., 2017). The exclusion criteria for the no-risk group were as follows: (1) minors younger than 9 years old or older than 12 years old, (2) psychological or psychiatric diagnosis, (3) special educational needs, (4) course repetition, (5) IQ < 80 or >115, and (6) clinical scores on BRIEF-2 Behavioral and Emotional Regulation Indexes at T1.

The inclusion criteria for the psychosocial risk (at-risk) group were as follows: (1) aged 9–12 years old, (2) diagnosed with psychosocial risk by the educational psychology services of the school, and (3) an IQ between 85 and <115. The exclusion criteria for at-risk group were as follows: (1) minors younger than 9 years old or older than 12 years old, (2) no diagnosis of psychosocial risk, and (3) IQ < 80 or >115.

The classification of students at psychosocial risk required a comprehensive assessment that integrated multiple sources of information and standardized tools. In this study, the identification of at-risk students was conducted in collaboration with the school’s educational guidance team, which had prior information provided by social workers. These professionals acted as intermediaries, facilitating communication between educational institutions and students’ socio-familial contexts.

To identify risk factors, specific criteria were established based on three fundamental dimensions. First, unmet basic needs were considered, including limited access to adequate medical care and the presence of malnutrition or untreated health conditions, as well as precarious housing situations, overcrowding, or residential instability. Additionally, food insecurity, inadequate diets, and insufficient access to nutritious food were assessed. Second, the family environment was analyzed, focusing on exposure to domestic violence, physical or emotional abuse, and neglect. The evaluation also considered inconsistent, authoritarian, or neglectful parenting practices; high levels of caregiver stress due to economic, employment, or mental health difficulties; and traumatic experiences such as bereavement, natural disasters, or violent events. Finally, educational and socio-educational deprivation was examined, including limited access to educational resources, insufficient school materials, a lack of digital learning devices, and low participation in extracurricular activities or enrichment programs. Families provided informed consent to participate in the study and to share information regarding the child’s diagnosis. However, access to detailed diagnostic reports is managed by the school’s guidance team, and the researchers did not consult these reports directly.

**Figure 1:**
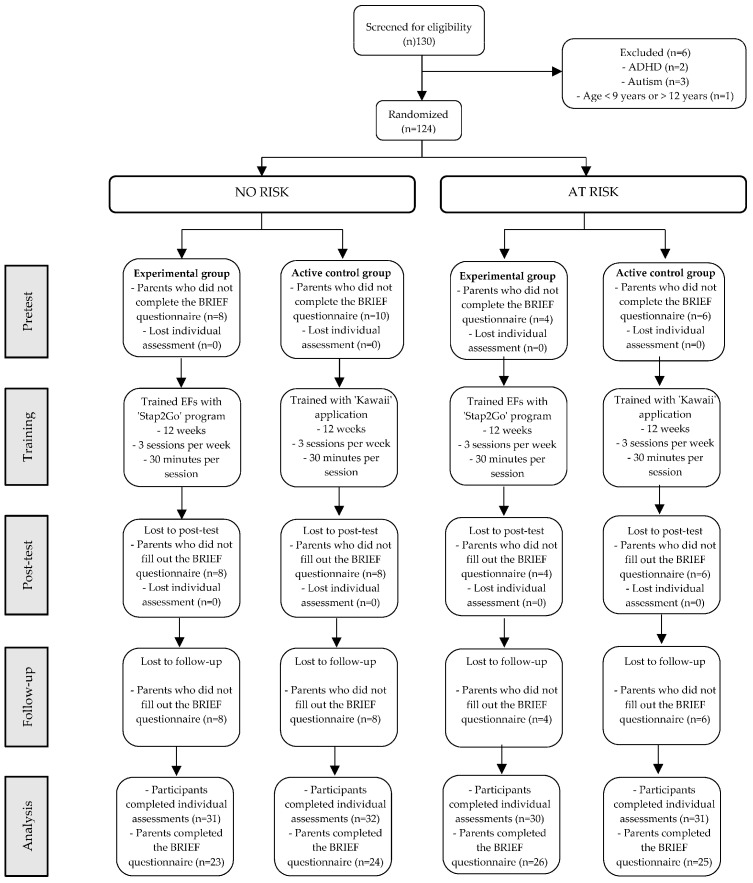
Participant flow diagram. Individual assessment implies FDT: Five Digit Test. WISC-V: Wechsler Intelligence Scale for Children Fifth Edition and RIST: Reynolds Intellectual Screening Test and academic performance (mathematics and Spanish language). BRIEF-2: Behavior rating inventory of executive functions.

Based on these criteria, social workers compiled detailed reports on students’ circumstances, which were then submitted to the school and the guidance department. This information enabled the classification and identification of students at psychosocial risk, ensuring a multidimensional approach to student support within educational settings. Figure 1 shows the sample sizes and dropouts as a function of a group.

**Figure 2:**
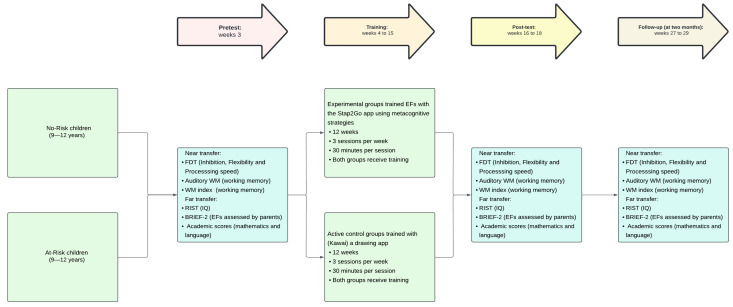
Illustration of the study design. Figure 2 legend: FDT: Five Digit Test. WISC-V: Wechsler Intelligence Scale for Children Fifth Edition; RIST: Reynolds Intellectual Screening Test; BRIEF-2: Behavior rating inventory of executive functions.

The independent variables include the interventions—STap2Go and Kawaii (Piu Piu Apps, 2023). The dependent variables of the study are (1) EFs (e.g., inhibition, WM, cognitive flexibility) and metacognition; (2) cognitive skills (e.g., intelligence, processing speed); (3) behavioral and emotional regulation; and (4) academic performance. Figure 2 clarifies the study design.

### 2.2. Assessments

Children were assessed before the training (T1), immediately after (T2), and two months after (T3). The evaluation was performed by a doctoral student and other qualified collaborating examiners in individual sessions. Families completed a questionnaire created ad hoc, as well as the BRIEF-2 questionnaire at T1, T2, and T3. Finally, the students’ tutors provided the participants’ academic scores (mathematics and language) at T1, T2, and T3.

- Background information questionnaire: This questionnaire was created ad hoc by the researchers. It was used to collect participant data related to the inclusion criteria and filled out by the children’s legal guardians. The questionnaire requested the following information: the participant’s age and sex; whether the child had received psychological or psychiatric treatment; whether the child had been diagnosed with specific educational needs; whether the child had repeated a school year; and whether the child had been identified by educational guidance services as being in a psychosocial risk situation. In cases where parents indicated that their children received psychological treatment or had specific educational needs, the information was assessed according to the study’s exclusion criteria. Children diagnosed with conditions such as ADHD, autism spectrum disorder, or significant learning disabilities were not eligible to participate. However, at this stage, no participants were excluded based on the information provided in the questionnaire.
- Five Digit Test (FDT) (Sedó, 2007): This assessment tool has different sections: Reading, Counting, Choice, and Alternation. In the Choice subscale, participants must count the numbers in a box instead of reading them. In the Alternation subscale, participants must change their strategy (from counting the numbers in a box to reading the numbers in it). The boxes in which the strategy is changed are marked by a blue frame. Additionally, the test includes subindices for “Reading time” and “Counting time”, which use the measure of processing speed. In the “Reading time” task, the child must read the numbers displayed in the box as quickly as possible. In the “Counting time” task, the child is required to count the number of asterisks in each box and say the total out loud. For example, if the card shows the stimulus “***”, the child should respond with “three”. We used inhibition and flexibility scores as variables of inhibition and flexibility. The inhibition score was determined by subtracting the reading time from the choice time. The flexibility score was determined by subtracting the reading time from the alternation time. Lower scores were associated with better results. The Spearman–Brown coefficient for this test ranges from 0.92 to 0.95.
- WM sub-index of the Wechsler Intelligence Scale (WISC-V): We used the Spanish adaptation of the WISC-V for children (2014), focusing on the WM index subtests: (1) Digits, which involves repeating digits in direct, reverse, and ascending order; (2) Drawing Span, requiring participants to recall and order drawings after brief exposure; and (3) Letters and Numbers, in which participants repeat numbers in ascending order and letters alphabetically after hearing a mixed series. The WM index is obtained through conversion to a composite index, which is calculated by summing the scaled scores of the Digits, Drawing Span, and Letter and Numbers subtests. The WM auditory index is obtained through conversion to a composite index, which is calculated by summing the scaled scores of the Digits and Letter and Numbers subtests. The Spanish adaptation for children WISC-V (Wechsler, 2015) was applied. Higher scores were associated with better results.
- Reynolds Brief Intelligence Test (RIST): RIST (Reynolds & Kamphaus, 2013) is a screening intelligence test that contains two subscales: Riddles, to assess verbal intelligence, and Categories, to assess non-verbal intelligence. The sum of both subscales determines the intelligence index (M = 100; SD = 15). Higher scores were associated with better results. The reliability for this test, based on Cronbach’s alpha, is 0.91.
- Behavior Rating Inventory of Executive Function (BRIEF-2) (Gioia et al., 2017): This standardized test for youth aged 5 to 18 focuses on assessing EFs with two versions, one for teachers and one for parents. For the purposes of this study, we focused on the family version. It is a Likert-type assessment in which the parent/guardian responds regarding frequency to a series of questions. Three main indices comprise the different clinical scales: the Behavioral Regulation Index, Emotional Regulation Index, and Cognitive Regulation Index. The Global index of executive function is made up of all three. It provides various scores related to EFs, such as inhibition, flexibility, self-control, WM, and cognitive regulation. Higher scores indicate problems or difficulties (T, typical scale; M = 50, SD = 10). This study used the Spanish adaptation (Gioia et al., 2017). This test has high reliability (based on Cronbach’s alpha M = 0.86).
- School performance: The numerical evaluations provided by the participants’ tutors in mathematics and language were used. The students’ academic performance ranged from 0 to 10. In the Spanish educational system at the primary education level, the homeroom tutor is the teacher responsible for a specific group of students. Academic performance in Spain is typically measured on a scale from 0 to 10, where 0 represents the lowest score and 10 represents the highest.

### 2.3. Interventions

Both groups received training sessions in groups of 10, conducted in the computer room during school hours and supervised by the researcher. The experimental group underwent training sessions lasting 12 weeks, following the STap2Go protocol (STap2Go, 2022). Students completed activities three times weekly in 30 min sessions on alternate days. When the participants enter the program (available at https://www.stap2go.es/, accessed on 14 November 2022), they access the “Neuronín World”. In this world, an avatar (Neuronín, a neuron-like character) will interact with different characters as participants progress through the circles on the board. Throughout the Neuronín World, a map-like structure with various elements designed along the session path (e.g., igloo, volcano…), participants will encounter characters with attentional and executive function difficulties. Neuronín will assist them by completing training sessions. By completing the activities, participants can advance along the path and meet new characters. Additionally, Neuronín earns a series of rewards based on the “energy” gained from performing the exercises. In Neuronín’s World, the following areas are available: (1) Neuronín’s House (where Neuronín can be taken care of), (2) the Gym (where Neuronín can perform exercises to train different abilities such as attention and executive functions; exercises appear randomly), (3) the Store (where Neuronín can be dressed), and (4) the Mini-game Lounge (where fun games can be played, which reward the completion of sessions). None of these areas are accessible until the participant completes the training sessions, and in this study, none of these features were used. Participants in the study only used the sessions path. On this world map, there is a path with circles (the protocol applied to participants), with each circle representing a session fixed by the protocol. By clicking on the first circle on the board, the STap2Go protocol begins. Each subsequent circle will unlock as the corresponding sessions are completed. The protocol consists of 100 exercises spread over 12 weeks (3 sessions per week on alternate days), carried out by clicking on each circle along the path in the established order. Each session includes 3 exercises with 2 repetitions of each. The program included go/no-go, stop-signal tasks, N-back tasks, and switching tasks, combined with metacognitive strategies provided before, during, and after the task (following a pre-designed decision tree) and after the tasks. The stimulus presentation (images and/or sounds) time was 1000–1500 ms, with a 500 ms window to respond. The average duration of each task was 2 min.

Participants received feedback in each trial, with the screen edge turning green for correct responses and red for mistakes. As participants progressed through the protocol, the difficulty level increased by combining different parameters (e.g., the number of distractors, n-back elements, the number of set shifts). Each session combined different task types in a balanced proportion through the sessions. All the instructions were specific for each task, resulting in around 100 different exercises ((e.g., (1) tap when you see something edible, (2) tap when you see a duck if the previous image was also a duck, (3) tap when you see a flower (...) now tap when you see a vegetable)). The metacognitive strategies were inspired by Efklides (2009) and designed by the creators of STap2Go. These strategies were validated in previous research (Rossignoli-Palomeque et al., 2019). The strategies focused on instruction comprehension, self-instructions, self-regulation, and performance supervision. Understanding instructions requires additional clarifications. For example, in the “tap when you see something red” exercise, before starting, the avatar asks (1) “When should we tap?”, prompting the user to tap when they see red, and (2) “What if something orange or pink appears?” to clarify that they should not tap if they do not see something red. These instructions help identify task demands. Self-instructions then guide the user during the task, with the avatar saying “Focus on the center of the screen to improve attention. What steps should we follow? First, wait, then observe, and finally, decide”. This helps direct attention and reduce impulsivity. As the user completes the exercises, the avatar offers targeted strategies based on specific errors (e.g., if the user taps impulsively, the avatar reminds them “Remember, first wait, then observe, then decide”), using a decision tree. After completing the tasks, the system reports the number and types of errors. The avatar then prompts the user to reflect on their mistakes (e.g., “I tapped when I shouldn’t have” or “I missed a required tap”) and suggests strategies for improvement. To motivate participants, they earn ‘energy’ points based on performance, which can be used for features such as avatar customization.

**Figure 3:**
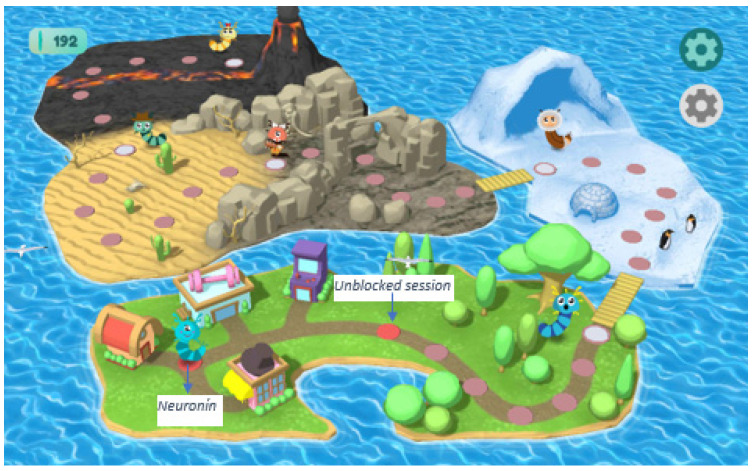
The “Neuronín” Word Map. Figure 3 legend: Stap2Go Screenshot. The Neuronín Word Map. Stap2Go (2022). Reproduced with permission.

**Figure 4:**
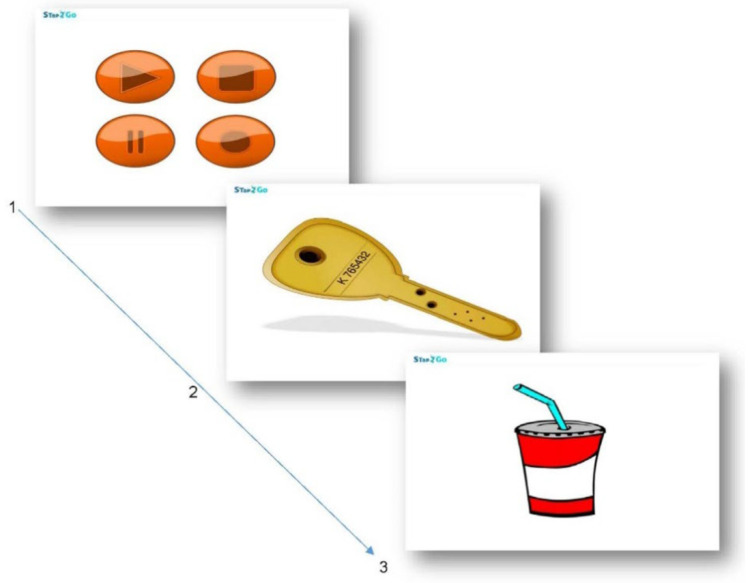
Stap2Go exercise example. Figure 4 legend: Stap2Go exercise example. Instruction: “Tap when you see something red”. In this case, the user has to tap in the third screen. Stap2Go (2022). Reproduced with permission.

**Figure 5:**
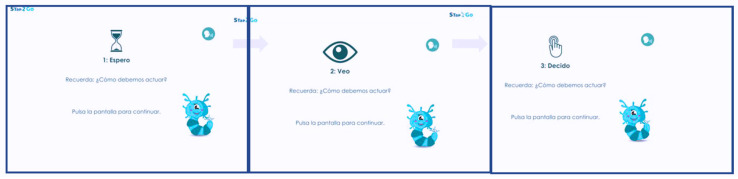
Example of Stap2Go strategy applied by avatar. Figure 5 legend: Self-control strategy in Stap2Go exercise applied when user taps impulsively: 1—“Wait” “Do you remember how we should act? 2—“See” “Do you remember how we should act? 3—“Decide” “Do you remember how we should act? Stap2Go (2022). Reproduced with permission.

All sessions were recorded through the application to ensure protocol fulfillment. Figure 3 shows the “Neuronín” Word Map, and Figure 4 shows an example of an EF task. Figure 5 exemplifies the metacognitive strategies applied.

The active control or placebo group received the same amount of intervention time as the experimental group but used another computer application (the Kawaii application). The Kawaii application (Piu Piu Apps, 2023) allows users to draw and create their compositions/designs. The rest of the conditions were the same for both groups (groups of 10 students using the application in the computer classroom, within school hours, supervised by the researcher). The researcher registered the training time and captured images of each participant.

### 2.4. Data Analysis

The sample size was estimated using G*Power 3.1.9.7 (Faul et al., 2020), based on the assumption that enrolling 140 participants would provide the trial with 95% power to detect an effect size of 0.35, which is considered a moderate effect size according to Cohen’s d conventions. Previous research by Rossignoli-Palomeque et al. (2020) reported an effect size of 0.29 in their research with the pilot version of the program used in this study. Although the effect size reported in this study was slightly lower, 0.35 was selected as a more conservative estimate for the power analysis to ensure the study’s robustness and ability to detect meaningful effects. First, we analyzed one-factor ANOVA at pretest (T1) to ensure no significant differences during the pretest among the groups.

To assess the intervention effects, a repeated-measures mixed ANCOVA 3 × 2 × 2 controlling for age was conducted with three factors included: time (three measurements: pretest, post-test, and follow-up), group (experimental and active control), and psychosocial risk condition (children at psychosocial risk and children without psychosocial risk). The main effects and inter-group differences were analyzed through Bonferroni post hoc tests. Effect size estimates were calculated using partial eta squared (*ηp*2), where *ηp*2 ≥ 0.01 is regarded as a small effect, *ηp*2 ≥ 0.06 as medium, and *ηp*2 ≥ 0.14 as large. If the one-way ANOVA indicated significant differences between groups during the pretest, we controlled for this variable in the ANCOVA. The p-value ranges from 0 to 1, with lower values indicating stronger evidence against the null hypothesis. The level of significance was set at *p* < 0.05. Hedges’ g was chosen as the effect size measure, as it is more appropriate than Cohen’s d when dealing with small samples (Hedges & Olkin, 1985). The interpretation of g is as follows: <0.2 = small effect, ≥0.5 = medium, and ≥0.8 = large (Cohen, 1988).

The analyses were conducted using JAMOVI 2.3.24 (The Jamovi Project, 2022). 

## 3. Results

### 3.1. Variables Considered to be Near Transfer Assessed Individually

**Figure 6:**
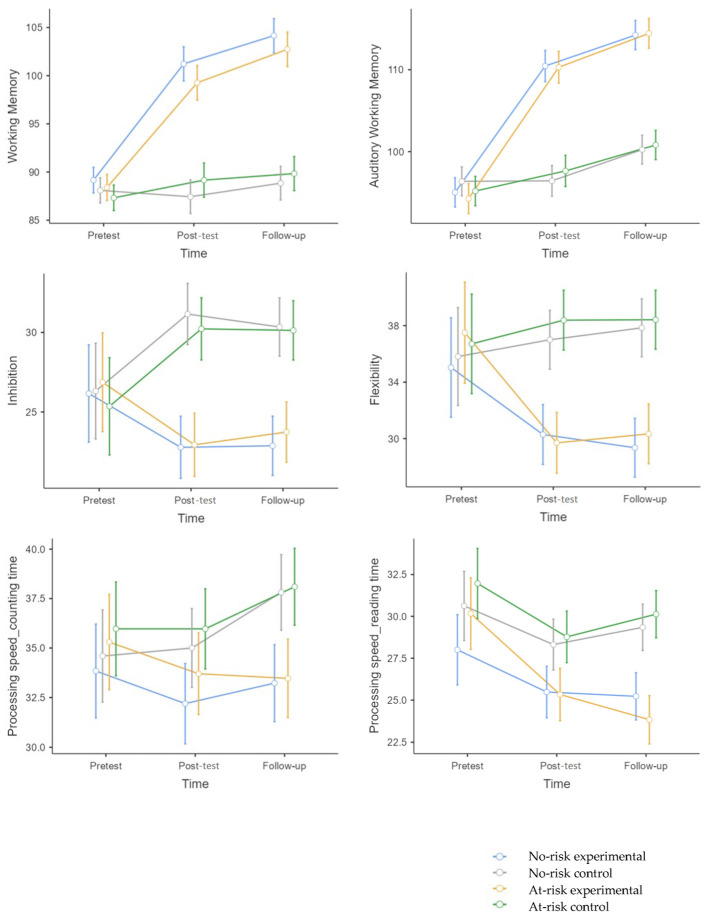
A Mean Chart of the variables considered to be near transfer assessed through individual assessments. Figure 6 legend: WM was assessed using the WISC-V; inhibition, flexibility, counting time, and reading time were assessed using the FDT.

Table 2 shows the mean and standard deviation of the variables considered to be near transfer (pretest, post-test, and follow-up) and the results of the ANOVA at T1. Figure 6 represents the significant near-transfer results after the pretest.

- WISC-V Working Memory Index

The main effect of time*group was significant (F (1,122) = 166, *p* < 0.001, η^2^p = 0.58). The experimental group (no-risk + at-risk) significantly improved at post-test (*p* < 0.001, g = 2.36) and follow-up (*p* < 0.001, g = 2.82) compared to the control group (no-risk + at-risk). The experimental group (no-risk + at-risk) improved at post-test (*p* < 0.001, g = 2.12) and follow-up (*p* < 0.001, g = 2.84) compared to pretest, and at follow-up compared to post-test (*p* < 0.001, g = 0.5).

The main effect of the time*group*risk condition was marginally significant (F (1,122) = 2.80, *p* = 0.06, η^2^p = 0.02). Children without psychosocial risk (no-risk): compared to the control group (no-risk), significant differences were found at post-test (*p* < 0.001, g = 2.3) and follow-up (*p* < 0.001, g = 2.7) in favor of the experimental group. Children at psychosocial risk (at-risk): compared to the control group (at-risk), significant differences were found at post-test (*p* < 0.001, g = 2.63) and follow-up (*p* < 0.001, g = 3.0) in favor of the experimental group. The experimental group (at-risk) significantly improved at follow-up compared to pretest (*p* = 0.02, g = 0.31). No significant differences were found between the experimental groups (no-risk vs. at-risk) at post-test (*p* = 1.00) or follow-up (*p* = 1.00).

- WISC-V Auditory Working Memory Index

The main effect of time*group was significant (F (1,122) = 166, *p* = < 0.001, η^2^p = 0.58). The experimental group (no-risk + at-risk) significantly improved at post-test (*p* < 0.001, g = 2.49) and follow-up (*p* = < 0.001, g = 2.75) compared to the control group (no-risk + at-risk). The experimental group (no-risk + at-risk) improved at post-test (*p* < 0.001, g = 2.85) and follow-up (*p* < 0.001, g = 3.89) compared to pretest, and at follow-up compared to post-test (*p* < 0.001, g = 0.67). The control group (no-risk + at-risk) significantly improved at post-test (*p* < 0.001, g = 0.25) and follow-up (*p* < 0.001, g = 0.94) compared to pretest. The main effect of time*group*risk condition was not significant (F (1,122) = 0.42, *p* = 0.65).

- FDT Inhibition

The main effect of time*group was significant (F (1,122) = 33, *p* = < 0.001, η^2^p = 0.22). The experimental group (no-risk + at-risk) significantly improved at post-test (*p* < 0.001, g = 1.42) and follow-up (*p* = < 0.001, g = 1.33) compared to the control group (no-risk + at-risk). The experimental group (no-risk + at-risk) significantly improved at post-test (*p* = 0.002, g = 0.53) and follow-up (*p* = 0.02, g = 0.45). The control group (no-risk + at-risk) significantly worsened at post-test (*p* = < 0.001, g = 0.65) and follow-up (*p* = < 0.001, g = 0.61). The main effect of the time*group*risk condition was not significant (F (1,122) = 0.04, *p* = 0.95).

- FDT Flexibility

The main effect of time*group was significant (F (1,122) = 18.91, *p* = < 0.001, η^2^p = 0.14). The experimental group (no-risk + at-risk) significantly improved at post-test (*p* = < 0.001, g = 1.29) and follow-up (*p* = < 0.001, g = 1.41) compared to the control group (no-risk + at-risk). The experimental group (no-risk + at-risk) significantly improved at post-test (*p* = < 0.001, g = 0.45) and follow-up (*p* = < 0.001, g = 0.48) compared to pretest. The main effect of the time*group*risk condition was not significant (F (1,122) = 0.72, *p* = 0.48).

- FDT Counting Time (Processing Speed)

The main effect of time*group was significant (F (1,122) = 12.14, *p* = < 0.001, η^2^p = 0.1). The experimental group (no-risk + at-risk) significantly improved at follow-up (*p* = < 0.001, g = 0.84) compared to the control group (no-risk + at-risk). The main effect of the time*group*risk condition was not significant (F (1,122) = 0.07, *p* = 0.92).

- FDT Reading Time (Processing Speed)

The main effect of time*group was significant (F (1,122) = 7.82, *p* = < 0.001, η^2^p = 0.06). The experimental group (no-risk + at-risk) significantly improved at post-test (*p* = 0.002, g = 0.72) and follow-up (*p* = < 0.001, g = 1.30) compared to the control group (no-risk + at-risk). The main effect of time*group*risk condition was not significant (F (1,122) = 1.82, *p* = 0.16).

### 3.2. Variables Considered to be Transferable in Participants’ Daily Lives Were Assessed Through Parent Questionnaires

**Figure 7:**
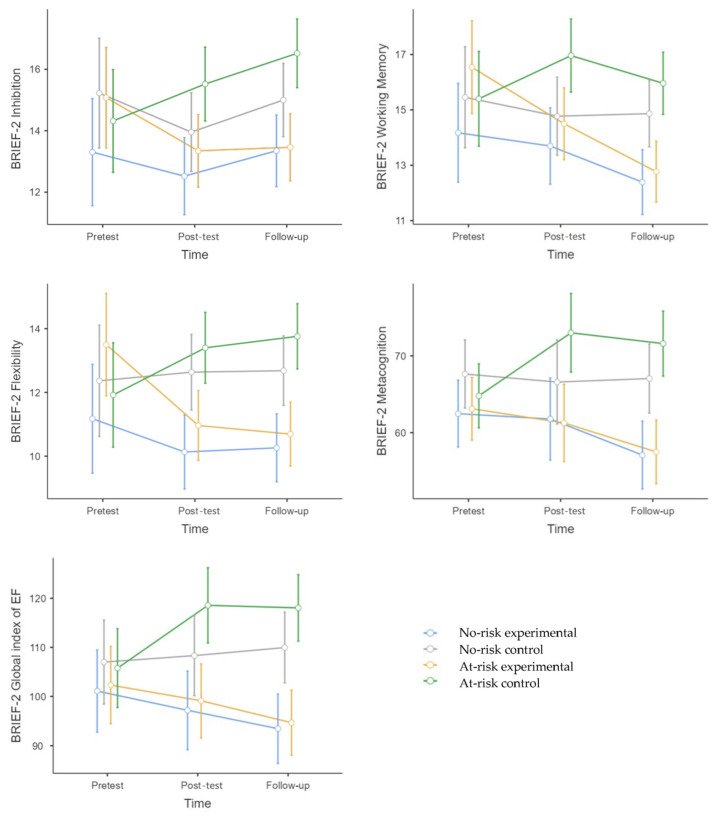
A Mean Chart of the variables considered to be transferable in participants’ daily lives with variables assessed through parent questionnaires. Figure 7 legend: All of these variables were assessed using BRIEF-2, completed by parents. Higher scores indicate greater difficulties in the respective domain.

Table 3 shows the average and standard deviation of the variables considered to be transferable in participants’ daily lives (pretest, post-test, and follow-up) as well as the results of the T1 ANOVA. Figure 7 shows the significant results after the pretest in the participants’ daily lives.

- BRIEF-2 Inhibition

The main effect of time*group was not significant (F (1,122) = 2.35, *p* = 0.09). Nevertheless, there was a significant effect for the time*group*risk condition (F (1,122) = 3.53, *p* = 0.03, η^2^p = 0.04). No-risk: Compared to the control group, no significant differences were found at post-test (*p* = 1.00) or follow-up (*p* = 1.00). At-risk: Compared to the control group, significant differences were found at follow-up (*p* = 0.013, g = 0.94) in favor of the experimental group. The difference between the experimental groups (no-risk vs. at-risk) was not significant at either post-test (*p* = 1.00) or follow-up (*p* = 1.00).

- BRIEF-2 Flexibility

The main effect of time*group was significant (F (1,122) = 7.39, *p* = < 0.001, η^2^p = 0.08). The experimental group (no-risk + at-risk) significantly improved at post-test (*p* = < 0.001, g = 0.90) and follow-up (*p* = < 0.001, g = 1.41) compared to the control group (no-risk + at-risk). The main effect of the time*group*risk condition was not significant (F (1,122) = 2.35, *p* = 0.09).

- BRIEF-2 Working Memory

The main effect of time*group was significant (F (1,122) = 4.33, *p* = 0.01, η^2^p = 0.05). The experimental group (no-risk + at-risk) significantly improved at follow-up (*p* = < 0.001, g = 1.01) compared to the control group (no-risk + at-risk). The experimental group (no-risk + at-risk) improved at follow-up compared to pretest (*p* = 0.003, g = 0.29), and compared to post-test (*p* = 0.003, g = 0.56). The main effect of the time*group*risk condition was not significant (F (1,122) = 2.46, *p* = 0.08).

- BRIEF-2 Metacognition Index

The main effect of time*group was significant (F (1,122) = 7.54, *p* = < 0.001, η^2^p = 0.08). The experimental group (no-risk + at-risk) significantly improved at post-test (*p* = 0.02, g = 0.64) and follow-up (*p* = < 0.001, g = 1.13) compared to the control group (no-risk + at-risk). The experimental group (no-risk + at-risk) improved at follow-up compared to pretest (*p* = 0.02, g = 0.58), and compared to post-test (*p* = 0.01, g = 0.41).

The main effect of the time*group*risk condition was marginally significant (F (1,122) = 3.00, *p* = 0.05, η^2^p = 0.03). No-risk: Compared to the control group, no significant differences were found at post-test (*p* = 1.00) or follow-up (*p* = 0.15). At-risk: Compared to the control group, significant differences were found at follow-up (*p* = < 0.001, g = 1.15) in favor of the experimental group. The difference between the experimental groups (no-risk vs. at-risk) was not significant at either post-test (*p* = 1.00) or follow-up (*p* = 1.00).

- BRIEF-2 Global Index of Executive Function

The main effect of time*group was significant (F (1,122) = 8.12, *p* = < 0.001, η^2^p = 0.08). The experimental group (no-risk + at-risk) significantly improved at post-test (*p* = 0.003, g = 0.80) and follow-up (*p* = < 0.001, g = 1.18) compared to the control group (no-risk + at-risk). The main effect of the time*group*risk condition was not significant (F (1,122) = 1.16, *p* = 0.31).

### 3.3. Variables Considered to be Far Transfer (Not Directly Trained)

**Figure 8:**
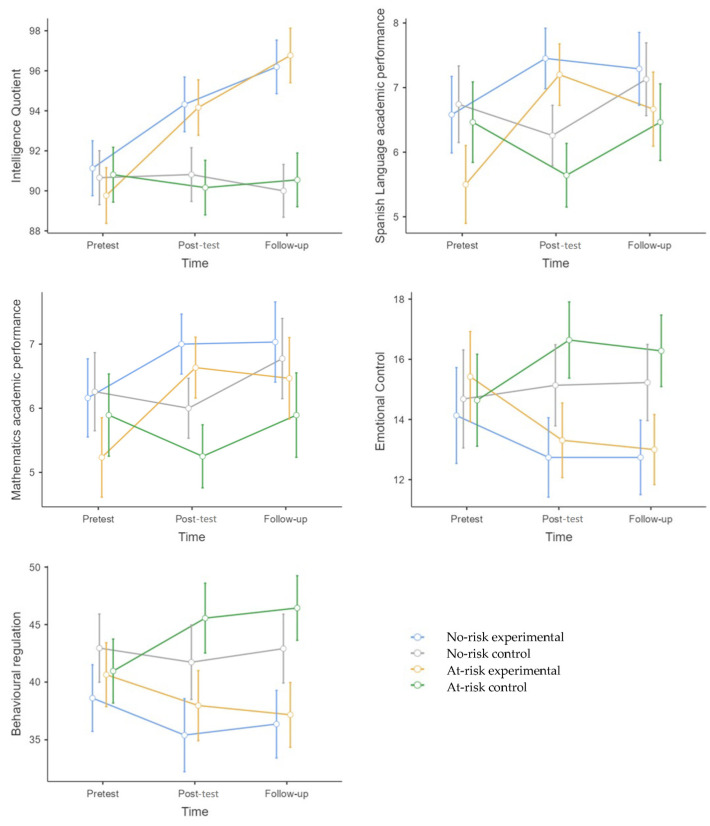
A Mean Chart of the variables considered to be far-transfer (variables not directly trained). Figure 8 legend: Intelligence Quotient was assessed using the RIST Index (M = 100, SD = 15). Academic performance was rated on a scale from 0 to 10. Emotional control and behavioral regulation were assessed using BRIEF-2, completed by the children’s families (higher scores on BRIEF-2 indicate greater difficulties in these domains).

Table 4 shows the average and standard deviation of the variables considered to be far transfer (pretest, post-test, and follow-up) as well as the results of the T1 ANOVA. Figure 8 illustrates the significant far-transfer results after the pretest.

- RIST Intelligence Index

The main effect of time*group was significant (F (1,122) = 65.98, *p* = < 0.001, η^2^p = 0.36). The experimental group (no-risk + at-risk) significantly improved at post-test (*p* = < 0.001, g = 0.98) and follow-up (*p* = < 0.001, g = 1.64) compared to the control group (no-risk + at-risk). The experimental group (no-risk + at-risk) improved at post-test (*p* = < 0.001, g = 0.87) and follow-up (*p* = < 0.001, g = 1.38) compared to pretest, and at follow-up (*p* = < 0.001, g = 0.49) compared to post-test. The main effect of the time*group*risk condition was not significant (F (1,122) = 1.74, *p* = 0.17).

- Spanish Language Academic Performance

The main effect of time*group was significant (F (1,122) = 74.42, *p* = < 0.001, η^2^p = 0.39). The experimental group (no-risk + at-risk) significantly improved at post-test (*p* = < 0.001, g = 0.98) compared to the control group (no-risk + at-risk). The experimental group (no-risk + at-risk) improved at post-test (*p* = < 0.001, g = 1.85) and follow-up (*p* = < 0.001, g = 0.58) compared to pretest. The control group significantly worsened at post-test (*p* = < 0.001, g = 0.41) compared to pretest.

The main effect of the time*group*risk condition was significant (F (1,122) = 7.42, *p* = < 0.001; η^2^p = 0.06). No-risk: Compared to the control group, significant differences were found at post-test (*p* = 0.01, g = 0.77) in favor of the experimental group. The experimental group significantly improved at post-test (*p* = < 0.001, g = 0.59) and follow-up (*p* = 0.013, g = 0.47) compared to pretest. At-risk: Significant differences were observed between the experimental and control groups at post-test (*p* = < 0.001, g = 0.77) in favor of the experimental group. The experimental group significantly improved at post-test (*p* = < 0.001, g = 1.17) and follow-up (*p* = < 0.001, g = 0.72) compared to pretest, and at follow-up (*p* = 0.005, g = 0.36) compared to post-test. The difference between the experimental groups (no-risk vs. at-risk) was not significant at either post-test (*p* = 1.00) or follow-up (*p* = 1.00).

- Mathematics Academic Performance

The main effect of time*group was significant (F (1,122) = 32.54, *p* = < 0.001, η^2^p = 0.22). The experimental group (no-risk + at-risk) significantly improved at post-test (*p* = < 0.001, g =.81) compared to the control group (no-risk + at-risk). The experimental group (no-risk + at-risk) improved at post-test (*p* = < 0.001, g = 0.76) and follow-up (*p* = < 0.001, g = 0.62) compared to pretest. The control group significantly worsened at post-test (*p* = < 0.001, g = 0.27) compared to pretest.

The main effect of the time*group*risk condition was significant (F (1,122) = 3.79, *p* = 0.02; η^2^p = 0.03). No-risk: Compared to the control group, there were no significant differences at post-test (*p* = 0.11) or follow-up. Nevertheless, the experimental group significantly improved at post-test (*p* = < 0.001, g = 0.61) and follow-up (*p* = 0.005, g = 0.55) compared to pretest. At-risk: The differences between the control and experimental groups were significant at post-test (*p* = 0.004, g = 0.98) in favor of the experimental group. The experimental group significantly improved at post-test (*p* = < 0.001, g = 0.91) compared to pretest. The difference between the experimental groups (no-risk vs. at-risk) was not significant at either post-test (*p* = 1.00) or follow-up (*p* = 1.00).

- BRIEF-2 Emotional Control

The main effect of time*group was significant (F (1,122) = 8.95, *p* = < 0.001, η^2^p = 0.09). The experimental group (no-risk + at-risk) significantly improved at post-test (*p* = < 0.001, g = 0.93) and follow-up (*p* = < 0.001, g = 1.00) compared to the control group (no-risk + at-risk). The main effect of the time*group*risk condition was not significant (F (1,122) = 1.25, *p* = 0.28).

- BRIEF-2 Behavioral Regulation Index

The main effect of time*group was significant (F (1,122) = 9.48, *p* = < 0.001, η^2^p = 0.10). The experimental group (no-risk + at-risk) significantly improved at post-test (*p* = < 0.001, g = 0.93) and follow-up (*p* = < 0.001, g = 1.15) compared to the control group (no-risk + at-risk).

The main effect of the time*group*risk condition was significant (F (1,122) = 3.43, *p* = 0.03; η^2^p = 0.04). No-risk: Compared to the control group, there were no significant differences at post-test (*p* = 0.45) or follow-up (*p* = 0.17). At-risk: The difference between the experimental and control groups was significant at post-test (*p* = 0.04, g = 0.86) and at follow-up (*p* = < 0.001, g = 1.13) in favor of the experimental group. The difference between the experimental groups (no-risk vs. at-risk) was not significant at either post-test (*p* = 1.00) or follow-up (*p* = 1.00).

## 4. Discussion

This study primarily aimed to assess the effects of a novel digital strategy-based method for the training of EFs, “STap2Go”, in children without psychosocial risk and with psychosocial risk (O1). Secondly, it aimed to examine whether the training impact differed between no-risk and at-risk children (O2), as might be expected according to the “compensation accounts” principle. Regarding this principle, those with greater needs (in this case, at-risk children) would benefit more from the training. It must be considered that Stap2Go is the first pure digital strategy-based intervention for EFs; thus, testing its effects is relevant. Our results support the idea presented by Niebaum and Munakata (2022), which claims that interventions designed to encourage children’s engagement with EFs may produce better outcomes than those simply aimed at improving EF capability.

In relation to O1, our results showed that the experimental group improved their EFs after the training, maintaining or even enhancing their results at follow-up (such as in WM, IQ, and metacognition). They improved in individual assessments of EFs (WM, auditory WM, inhibition, and cognitive flexibility) and processing speed (counting time and reading time). Moreover, there was transfer in the children’s daily lives, as observed in the family environment (global index of EFs, flexibility, metacognition, emotional control, and behavioral regulation). Unlike other process-based interventions in which children only improved in term of their EFs (Diamond et al., 2007; Liu et al., 2015; Zhao et al., 2018), our results also indicated transfer in terms of variables that affect children’s daily lives. Moreover, there was also a far transfer in IQ, mathematics, Spanish language academic performance, emotional control, and behavioral regulation. In addition, the effect size for most of the effects ranged from medium to large, which reinforces the impact of this training. To our knowledge, only two other studies have used digital EF strategy-based interventions on no-risk children (Jones et al., 2020; Pozuelos et al., 2018), finding stronger benefits in mathematics academic performance and IQ gains for those children who received the EF + metacognitive training. Previous studies with children in disadvantaged situations (or at-risk), such as those with special needs (Partanen et al., 2015) or children from disadvantaged backgrounds (Kubota et al., 2023), only found near-transfer effects. According to Zuber et al. (2022), WM training produces the largest performance improvements compared to other core elements of EFs. Our study, in contrast to those of Partanen et al. (2015) and Kubota et al. (2023), trained not only WM but also the main EF components, and this method seems to produce more benefits than WM alone. In the case of children with behavioral problems, Graziano and colleagues (2016) found stronger benefits for those children who received training with additional strategies over EFs, academic performance, emotional knowledge, and regulation. To our knowledge, this is the only study to find transfer in domains similar to those used in our study. Nevertheless, we must consider that the strategies were provided by trained parents. This variable could extend beyond the results of this study, as parents’ techniques and styles impact children’s behavior (Grolnick & Ryan, 1989; Sarwar, 2016). Our study is the only pure digital EF strategy-based intervention to produce such transfer effects without the influence of a human instructor.

It is remarkable that IQ, WM, and metacognition continue to improve at follow-up, suggesting a lasting training effect on these areas. To our knowledge, previous studies using digital training interventions have typically reported persistence of training effects, but not further improvement at follow-up assessments. However, a meta-analysis of the long-term effects of metacognitive strategies on academic performance by De Boer et al. (2018) showed that the effect of metacognition on student performance slightly increased from post-test to follow-up. We hypothesize that the incorporation of metacognitive strategies within the Stap2Go intervention played a central role in this sustained improvement. By promoting reflective thinking and self-regulation, participants may have developed greater awareness of their cognitive processes, which could have led to more effective adaptation and application of cognitive strategies to new tasks, even beyond the training period. Previous academic literature suggests the robust impact of procedural metacognition on various cognitive domains (De Boer et al., 2018; Pozuelos et al., 2018; Veenman et al., 2006; Zimmerman, 2002). Studies have consistently shown that metacognitive training, particularly that focused on strategy use and self-monitoring, can improve problem-solving abilities, cognitive flexibility, and the regulation of attention and memory (Veenman et al., 2006; Zimmerman, 2002). Notably, metacognitive strategies support not only the optimization of learning but also the consolidation and application of cognitive skills over time, commonly reflected in academic performance (De Boer et al., 2018). Thus, we consider that the sustained gains observed in IQ, WM, and metacognition could reflect the lasting influence of metacognitive training, where participants not only retained but also continued to refine their cognitive abilities long after the intervention ended. However, further studies are needed to address this new line of digital strategy-based training to better understand its long-term impact and the underlying mechanisms involved.

Our results support the transfer effects of EF strategy-based interventions. In contrast to the lack of transfer effects observed in this domain through process-based interventions (Melby-Lervåg et al., 2016; Rossignoli-Palomeque et al., 2018; Sala & Gobet, 2019; Simons et al., 2016; Takacs & Kassai, 2019), Stap2Go produces transfer effects on the emotional control and behavioral regulation of children. We believe this is due to training directed toward self-regulation, which is strongly related to emotional regulation (Diamond, 2013; Hofmann et al., 2012; Morgan & Lilienfeld, 2000; Riccio et al., 2011; Schoemaker et al., 2012; Séguin, 2009; Séguin et al., 1999). The transfer effects of Stap2Go align with the demonstrated relationship between EFs and intelligence (Ardila et al., 2000; Friedman et al., 2006; Rossignoli-Palomeque et al., 2018; Wang et al., 2021) and academic performance (Best et al., 2011; Cheung & Chan, 2022; Peng et al., 2015). Finally, a connection between EFs and emotional and behavioral regulation has also been established in several studies (Diamond, 2013; Hofmann et al., 2012; Morgan & Lilienfeld, 2000; Riccio et al., 2011; Schoemaker et al., 2012; Séguin, 2009; Séguin et al., 1999).

Our study results exceed the findings from the pilot study of Rossignoli-Palomeque et al. (2020), in which the strategies were facilitated by an instructor, and interventions were only assessed in no-risk children. In the present study, however, the latest version of the program was used, in which an avatar facilitates the strategies according to the user’s performance. This novel approach may promote the emergence of other digital strategy-based interventions, considering that process-based interventions primarily produced near transfer (Melby-Lervåg et al., 2016; Rossignoli-Palomeque et al., 2018; Sala & Gobet, 2019; Simons et al., 2016; Takacs & Kassai, 2019). Furthermore, the Stap2Go intervention may prevent the detriment suffered by children in conditions of psychosocial vulnerability, as the experimental group improved in most of the domains assessed, while the control group deteriorated over time.

Finally, we must also discuss two unexpected results. A positive effect was found in both groups (experimental and control) after the interventions. The controls also improved at post-test and follow-up in auditory WM. Although the effect size was larger for the experimental group (post-test, *g* = 0.67) compared to the control group (post-test, *g* = 0.25), we consider this unexpected result a limitation, possibly due to a practice effect. Future studies may consider using tests or tasks with parallel forms to avoid this limitation. Still, we must consider that there was one negative effect for the control group. In the FDT inhibition, the control group (no-risk + at-risk) significantly worsened at post-test and follow-up. Despite significant statistical differences, the increasing scores over time suggest a deterioration in performance, indicating that the control group, which did not receive the intervention, showed a decline in their inhibition skills over time.

To evaluate a possible difference in training impact considering the presence or absence of a psychosocial risk condition (O2), we used a 3 × 2 × 2 triple interaction ANOVA. In most cases, this interaction was not significant, which suggests that the impact of the training did not vary importantly between the groups with and without psychosocial risk. Overall, the Stap2Go intervention seems effective in both conditions; however, some effects were more pronounced in children with psychosocial risk. The time*group*risk condition interaction showed that in some variables (BRIEF-2 Inhibition, BRIEF-2 Metacognition, and, BRIEF-2 Behavioral Regulation, mathematics and language academic performance), children with psychosocial risk (at-risk) experienced more noticeable improvements compared to children without psychosocial risk (no-risk). These results suggest that the intervention was especially beneficial for children in at-risk situations in those variables. Our results support the ‘compensation accounts’ principle, which posits that individuals with greater needs benefit more from training (Diamond, 2013; Diamond & Ling, 2019; Karbach, 2015; Smid et al., 2020). De Boer et al.’s (2018) meta-analysis of the long-term effects of 48 metacognitive strategy-based instruction interventions showed that children with difficulties, such as those from low-socioeconomic-status (SES) backgrounds, experienced a greater benefit from metacognitive training. These low-SES students, who can be considered part of the ‘at-risk’ group, showed the most improvement in performance from post-test to follow-up in academic performance, supporting the idea that children with greater needs may benefit more from metacognitive training, as proposed by compensation accounts. Our results align with the scientific literature on this topic.

In addition, although no statistically significant results were found for the at-risk control group, a trend toward worsening scores was observed across several areas over time, particularly in cognitive and emotional regulation, as well as academic performance. This tendency was not found in the no-risk control group, suggesting that the at-risk condition may play a role in the observed decline. Specifically, the at-risk control group showed increasing scores in BRIEF-2 (Inhibition, Behavioral Regulation, Emotional Control, Flexibility, and Global Executive Function), indicating greater difficulties in these areas over time according to the family environment. Academic performance, both in Spanish and mathematics, also showed a slight decline over time. These findings suggest that while the changes were not statistically significant, the at-risk control group exhibited a trend toward worsening scores across these areas, highlighting the need for targeted interventions in this population. (See Table 2, Table 3 and Table 4 and Figure 5, Figure 6 and Figure 7). This deterioration seems to occur without receiving a specific intervention in EFs. These results highlight the relevance of this intervention for a particularly vulnerable group. At-risk children may deteriorate if no adequate intervention is carried out. Our results support the notion that children from disadvantaged socio-educational conditions benefit the most from this type of intervention (Diamond, 2013; Diamond & Ling, 2019).

Regarding study design, contrary to others (Graziano & Hart, 2016; Partanen et al., 2015), our study includes an active control group. This approach reduces the likelihood of committing a Type I error and strengthens the validity of the results (Field, 2013). Furthermore, our study includes a 2-month follow-up, during which most of the effects were maintained. This methodological approach represents a significant strength of our study, as it enhances the robustness and generalizability of the findings. Although other strategy-based intervention studies include follow-ups (Graziano & Hart, 2016; Kubota et al., 2023; Jones et al., 2020), the maintained effects are only near transfer (except for Graziano and colleagues’ study [2016], which we have already discussed).

Finally, this study faced the limitation of sample loss, as not all families provided the questionnaires at different evaluation moments. Additionally, the consideration of psychosocial risk is very broad, making it a heterogeneous group. In future studies, we would like to perform a more detailed analysis according to specific risk conditions. As a future direction, we would extend the study to other developmental stages to determine whether there are greater or lesser benefits depending on participants’ ages. Finally, we intend to compare a group receiving the Stap2Go intervention with another group receiving the Stap2Go intervention without metacognitive strategies to assess the impact of the strategies on the results. Additionally, a latent-change score study will help us better understand the influence of metacognition on training effects.

The findings of this study have significant implications for both research and educational practice. The notable improvements observed in executive functions, IQ, academic performance, and emotional and behavioral regulation indicate that STap2Go is an effective digital training strategy for enhancing cognitive and academic outcomes in children. Importantly, the stronger effects seen in children at psychosocial risk suggest that this type of intervention can serve as a valuable tool to help mitigate the cognitive and academic challenges associated with socio-educational vulnerability.

Moreover, the continued improvements in IQ, metacognition, and working memory observed at follow-up provide evidence for the long-term benefits of digital cognitive training. In contrast, the decline seen in the control group of at-risk children highlights the urgent need for early interventions to prevent cognitive and academic deterioration in vulnerable populations.

These findings underscore the importance of integrating strategy-based digital cognitive training programs into educational settings, especially for children facing socio-economic adversity. Future research should investigate the scalability of this intervention, its effectiveness across diverse populations, and its potential integration with broader educational and psychological support systems.

## 5. Conclusions

The experimental group significantly improved in individual assessments of basic EFs, IQ, processing speed (counting time and reading time), and EFs according to family assessments compared to the controls. Furthermore, the experimental groups improved in terms of emotional and behavioral regulation, and academic performance (i.e., language and mathematics), after the intervention. These results were maintained at follow-up. Moreover, IQ, metacognition and WM continued to improve at follow-up, suggesting a lasting training effect on these areas.

Generally, the control group did not improve, and there was even a trend of deterioration in some areas for the at-risk control group according to their family environment (i.e., in inhibition, flexibility, global index of EFs, metacognition, and behavioral regulation) at post-test and/or follow-up, and over academic performance. This suggests that this group appears to be more vulnerable than the no-risk group.

Although the intervention benefits both no-risk and at-risk children, some effects were more pronounced in children with psychosocial risk. Specifically on BRIEF-2 (Inhibition, Metacognition, Behavioral Regulation) and mathematics and Spanish language academic performance at-risk children experienced more noticeable improvements compared to no-risk children.

## Figures and Tables

**Table 1:** Sociodemographic characteristics of the participants.

		RIST	Gender F/M	Age
Pretest	No-risk experimental group	91.1 (4.76)	14/17	133 (12.9)
	No-risk control group	90.7 (3.70)	12/20	131 (6.96)
	At-risk experimental group	89.8 (3.22)	14/16	135 (8.76)
	At-risk control group	90.8 (3.53)	21/10	133 (10.3)
	*F*(3.120)	0.69 (*p* = 0.057)		1.13 (*p* = 0.34)
Post-test	No-risk experimental group	94.3 (5.06)	14/17	136 (12.9)
	No-risk control group	90.7 (3.17)	12/20	135 (6.96)
	At-risk experimental group	94.2 (4.04)	14/16	138 (8.75)
	At-risk control group	90.8 (3.53)	21/10	133 (10.3)
	*F*(3.120)	10.1 (*p* < 0.001)		1.14
Follow-up	No-risk experimental group	96.2 (5.36)	14/17	140 (12.9)
	No-risk control group	90.0 (2.62)	12/20	138 (6.94)
	At-risk experimental group	96.8 (3.54)	14/16	142 (8.76)
	At-risk control group	90.5 (3.00)	21/10	90.5 (3.00)
	*F*(14.5)	32.4 (*p* < 0.001)		1.13

Table 1 legend: F = female; M = male; RIST: Reynolds Intellectual Screening Test; Age = participants’ ages in terms of month.

**Table 2:** The mean and standard deviation of the variables considered to be near transfer (pretest, post-test, and follow-up) and the results of the ANOVA at T1 for no-risk and at-risk children.

		No-Risk Experimental Group	No-Risk Control Group	No-Risk ANOVA t1 *p*-Value	At-Risk Experimental Group	At-Risk Control Group	At-Risk ANOVA t1 *p*-Value
		*M* (*SD*)	*M* (*SD*)		*M* (*SD*)	*M* (*SD*)	
WISC-V Working Memory Index	Pretest	89.2 (4.69)	88.1 (3.45)	0.31	88.4 (2.92)	87.3 (3.61)	0.20
	Post-test	101.2(8.10)	87.4 (2.34)		99.3 (4.28)	89.2 (3.26)	
	Follow-up	104.2 (7.53)	88.8 (2.77)		102.7 (4.23)	89.8 (4.15)	
WISC-V Auditory Working Memory Index	Pretest	95.0 (4.17)	96.4 (5.36)	0.27	94.3 (5.07)	95.2 (5.45)	0.49
	Post-test	110.5 (7.39)	96.4 (4.75)		110.3 (4.88)	97.6 (3.72)	
	Follow-up	114 (6.56)	100 (4.45)		114 (4.02)	101 (4.66)	
FDT Inhibition	Pretest	26.2 (8.43)	26.3 (9.53)	0.95	26.9 (8.37)	25.4 (7.94)	0.47
	Post-test	22.8 (5.96)	31.2 (5.52)		22.9 (4.06)	30.2 (6.20)	
	Follow-up	22.9 (5.80)	30.3 (4.49)		23.7 (5.04)	30.1 (5.55)	
FDT Flexibility	Pretest	35.0 (9.90)	35.8 (9.44)	0.75	37.5 (9.55)	36.7 (10.7)	0.76
	Post-test	30.3 (6.47)	37.0 (6.14)		29.7 (5.90)	38.4 (5.28)	
	Follow-up	29.4 (5.74)	37.8 (6.07)		30.3 (5.80)	38.4 (5.82)	
FDT Counting Time	Pretest	33.8 (6.55)	34.6 (5.29)	0.62	35.3 (6.19)	36.0 (8.29)	0.72
	Post-test	32.2 (4.91)	35.0 (2.88)		33.7 (5.47)	36.0 (8.26)	
	Follow-up	33.2 (5.20)	37.8 (4.89)		33.5 (5.16)	38.1 (6.49)	
FDT Reading Time	Pretest	28.0 (4.64)	30.6 (5.32)	0.04	30.2 (6.75)	32.0 (6.68)	0.3
	Post-test	25.5 (3.78)	28.3 (3.23)		25.3 (3.11)	28.8 (6.38)	
	Follow-up	25.2 (3.17)	29.3 (3.43)		23.8 (2.67)	30.1 (5.82)	

Table 2 legend: FDT: Five Digit Test; WISC-V: Wechsler Intelligence Scale for Children Fifth Edition.

**Table 3:** The average and standard deviation of the variables considered to be transferable in participants’ daily lives (pretest, post-test, and follow-up) for each dependent variable, as well as the results of the T1 ANOVA for no-risk and at-risk children.

		No-Risk Experimental Group	No-Risk Control Group	No-Risk ANOVA t1 *p*-Value	At-Risk Experimental Group	At-Risk Control Group	At-Risk ANOVA t1 *p*-Value
		*M* (*SD*)	*M* (*SD*)		*M* (*SD*)	*M* (*SD*)	
BRIEF-2 Inhibition	Pretest	13.3 (2.57)	15.2 (3.25)	0.03	15.1 (6.23)	14.3 (3.50)	0.59
	Post-test	12.5 (1.81)	13.9 (3.03)		13.3 (3.22)	15.5 (3.68)	
	Follow-up	13.3 (1.70)	15.0(2.68)		13.5 (2.73)	16.5 (3.64)	
BRIEF-2 Flexibility	Pretest	11.2 (2.35)	12.4 (2.50)	0.11	13.5 (6.54)	11.9 (3.17)	0.28
	Post-test	10.1 (2.05)	12.8 (2.80)		11.0 (2.96)	13.4 (3.15)	
	Follow-up	10.3 (1.81)	13.0 (3.05)		10.7 (2.54)	13.8 (2.85)	
BRIEF-2 Working Memory	Pretest	14.2 (2.71)	15.5 (2.22)	0.32	16.5 (7.15)	15.4 (2.63)	0.45
	Post-test	13.7 (2.40)	14.8 (3.84)		14.5 (3.54)	17.0 (3.55)	
	Follow-up	12.4 (1.34)	15.0 (2.71)		12.8 (2.94)	16.0 (3.81)	
BRIEF-2 Metacognition Index	Pretest	62.5 (10.09)	67.6 (9.62)	0.09	63.1 (10.86)	64.8 (11.16)	0.58
	Post-test	61.8 (11.7)	66.9 (14.5)		61.3 (11.9)	73.0 (13.9)	
	Follow-up	57.1 (6.86)	67.6 (11.20)		57.5 (9.70)	71.6 (14.12)	
BRIEF-2 Global Index of Executive Functions	Pretest	101 (15.9)	107 (25.6)	0.36	102 (19.4)	106 (19.3)	0.53
	Post-test	97.2 (14.2)	109.0 (20.9)		99.1 (19.1)	118.6 (22.2)	
	Follow-up	93.4 (9.55)	111.0 (17.70)		94.7 (15.93)	118.0 (22.62)	

Table 3. BRIEF-2: Behavior rating inventory of executive functions.

**Table 4:** Means and standard deviation of the variables considered to be far transfer (pretest, post-test, and follow-up) as well as the results of the T1 ANOVA for no-risk and at-risk children.

		No-Risk Experimental Group	No-Risk Control Group	No-Risk ANOVA t1 *p*-Value	At-Risk Experimental Group	At-Risk Control Group	At-Risk ANOVA t1 *p*-Value
		*M* (*SD*)	*M* (*SD*)		*M* (*SD*)	*M* (*SD*)	
RIST Intelligence Index	Pretest	91.1 (4.76)	38.6 (6.60)	0.66	89.8 (3.22)	90.0 (3.53)	0.23
	Postest	94.3 (5.06)	35.4 (3.38)		94.2 (4.04)	90.2 (2.68)	
	Follow-up	96.2 (5.36)	36.3 (3.51)		96.8 (3.54)	90.5 (3.00)	
Spanish Language academic performance	Pretest	6.58 (1.52)	6.74 (2.18)	0.74	5.50 (1.61)	6.46 (1.14)	0.01
	Postest	7.45 (1.39)	6.31 (1.53)		7.20 (1.24)	5.61 (1.28)	
	Follow-up	7.29 (1.49)	7.19 (1.71)		6.67 (1.60)	6.39 (1.56)	
Mathematics academic performance	Pretest	6.16 (1.55)	6.26 (2.16)	0.84	5.23 (1.65)	5.89 (1.34)	0.10
	Postest	7.00 (1.13)	6.06 (1.58)		6.63 (1.35)	5.32 (1.30)	
	Follow-up	7.03 (1.60)	6.84 (1.89)		6.47 (1.74)	5.90 (1.85)	
BRIEF-2 Emotional Control	Pretest	14.1 (2.97)	14.7 (2.66)	0.51	15.4 (5.55)	14.6 (3.21)	0.54
	Postest	12.7 (1.39)	15.4 (2.84)		13.3 (3.07)	16.6 (4.53)	
	Follow-up	12.7 (1.60)	15.5 (2.92)		13.0 (2.68)	16.3 (4.16)	
BRIEF-2 Behavioral Regulation Index	Pretest	38.6 (6.60)	43.0 (6.47)	0.03	40.8 (5.61)	41.0 (8.76)	0.12
	Postest	35.4 (3.38)	42.1 (7.47)		37.8 (8.14)	45.6 (9.56)	
	Follow-up	36.3 (3.51)	43.5 (7.29)		37.2 (6.97)	46.4 (9.12)	

Table 4 legend: RIST: Reynolds Brief Intelligence Index. Curricular subjects: Spanish Language academic performance and Mathematics academic performance. BRIEF: Behavior rating inventory of executive functions.

## Data Availability

The authors will provide data under request to any interested researchers.

## Abbreviations

The following abbreviations are used in this manuscript:

No-risk	Children without psychosocial risk
At-risk	Children at psychosocial risk
EFs	Executive functions
WM	Working memory
